# Glycyrrhizic Acid for COVID-19: Findings of Targeting Pivotal Inflammatory Pathways Triggered by SARS-CoV-2

**DOI:** 10.3389/fphar.2021.631206

**Published:** 2021-06-09

**Authors:** Wenjiang Zheng, Xiufang Huang, Yanni Lai, Xiaohong Liu, Yong Jiang, Shaofeng Zhan

**Affiliations:** ^1^ The First Clinical Medical School, Guangzhou University of Chinese Medicine, Guangzhou, China; ^2^ The First Affiliated Hospital, Guangzhou University of Chinese Medicine, Guangzhou, China; ^3^ Lingnan Medical Research Center, Guangzhou University of Chinese Medicine, Guangzhou, China; ^4^ Guangzhou University of Chinese Medicine, Guangzhou, China; ^5^ Shenzhen Hospital of Integrated Traditional Chinese and Western Medicine, Shenzhen, China

**Keywords:** coronavirus disease 2019, SARS-CoV-2, glycyrrhizic acid, network pharmacology, inflammatory pathways

## Abstract

**Background:** Coronavirus disease 2019 (COVID-19) is now a worldwide public health crisis. The causative pathogen is severe acute respiratory syndrome coronavirus 2 (SARS-CoV-2). Novel therapeutic agents are desperately needed. Because of the frequent mutations in the virus and its ability to cause cytokine storms, targeting the viral proteins has some drawbacks. Targeting cellular factors or pivotal inflammatory pathways triggered by SARS-CoV-2 may produce a broader range of therapies. Glycyrrhizic acid (GA) might be beneficial against SARS-CoV-2 because of its anti-inflammatory and antiviral characteristics and possible ability to regulate crucial host factors. However, the mechanism underlying how GA regulates host factors remains to be determined.

**Methods:** In our report, we conducted a bioinformatics analysis to identify possible GA targets, biological functions, protein-protein interactions, transcription-factor-gene interactions, transcription-factor-miRNA coregulatory networks, and the signaling pathways of GA against COVID-19.

**Results:** Protein-protein interactions and network analysis showed that ICAM1, MMP9, TLR2, and SOCS3 had higher degree values, which may be key targets of GA for COVID-19. GO analysis indicated that the response to reactive oxygen species was significantly enriched. Pathway enrichment analysis showed that the IL-17, IL-6, TNF-α, IFN signals, complement system, and growth factor receptor signaling are the main pathways. The interactions of TF genes and miRNA with common targets and the activity of TFs were also recognized.

**Conclusions:** GA may inhibit COVID-19 through its anti-oxidant, anti-viral, and anti-inflammatory effects, and its ability to activate the immune system, and targeted therapy for those pathways is a predominant strategy to inhibit the cytokine storms triggered by SARS-CoV-2 infection.

## Introduction

Severe acute respiratory syndrome coronavirus 2 (SARS-CoV-2), the pathogen of coronavirus disease 2019 (COVID-19), is the greatest public health crisis nowadays. According to the World Health Organization (https://covid19.who.int/), as of January 21, 2020, there were 95,612,831 confirmed cases worldwide and 2,066,176 confirmed deaths. Novel therapeutic agents and vaccines are desperately needed to address the COVID-19 crisis. Many studies conducted in clinical settings have shown that traditional Chinese medicine plays an essential role in the treatment of COVID-19. Seen through the lens of conventional Western medicine, it has offered new medical solutions to control the disease (Huang et al., 2020). Many chemical components of Chinese medicine have been confirmed to provide specific health effects, including anti-inflammatory and antiviral activity *in vitro* and *in vivo* (Chu et al., 2020). Glycyrrhizic acid (GA) might be beneficial against SARS-CoV-2 for its anti-inflammatory and antiviral characteristics (Siddiqui et al., 2020). GA, a triterpene saponin, is considered the principal component in *Glycyrrhizae Radix et Rhizoma* with various biological functions and pharmacological effects, including a wide spectrum of anti-inflammatory activity, antiviral activity and immunomodulatory effects. Several of the biochemical, molecular, and cellular mechanisms underlying the action of GA has also been explored in experimental studies. GA has been shown to abolish the expression of ICAM-1 upregulation, reduce the amounts of TNF-α and IL-1β in the plasma, reduce STAT-3 activation, and suppress NF-κB activation in inflammation (Di Paola et al., 2009).

Particularly, one study in *Nature* indicated that GA inhibits viral growth and inactivates viral particles (Pompei et al., 1979), in addition to inhibiting virus replication. A study in *Lancet* revealed that GA was the most active in inhibiting replication of the SARS-associated virus especially the adsorption and penetration of viruses early in the replication cycle (Cinatl et al., 2003). A study in *Phytomedicine* has shown that GA exerted inhibitory activity against the spike protein of SARS-CoV-2 (Yu et al., 2020). Underlying mechanisms of GA against COVID-19 has also been explored in several bioinformatic studies (Chen et al., 2020a; Li R. et al., 2020; Patil et al., 2021). A systematic review of five retrospective cohort studies has indicated that GA could be an optional therapeutic strategy for SARS-CoV-2 infections (Li H. et al., 2020). Importantly, there is one on-going pilot, non-randomized clinical study with GA as complementary medicine in patients with COVID-19 (NCT04487964).

GA induces cholesterol-dependent disintegration of lipid rafts at the membrane level, which is important for hindering the entry of coronaviruses into cells. At the intracellular and circulating levels, GA can capture the high mobility group box 1 protein (HMGB1), thereby blocking the alarm function itself (Bailly and Vergoten, 2020). Researchers performed genome-wide CRISPR screens with of SARS-CoV-2, and they demonstrated that HMGB1 regulated ACE2 transcription, which is indispensable for the entry of SARS-CoV-2 (Wei et al., 2020). In terms of clinical evidence, it has been reported that elevated serum HMGB1 on admission is associated with poor clinical prognosis of COVID-19 patients (Chen et al., 2020b). Extracellular HMGB1 may be an attractive potential therapeutic target for severe lung inflammation such as COVID-19 (Andersson et al., 2020). In COVID-19 patients, the host response failed to launch a strong IFN response while a waning immune response would enable sustained viral replication and further simultaneously induced high levels of chemokines (Blanco-Melo et al., 2020). And this is closely related to the severity of SARS-CoV-2 infection. Excitingly, GA contributes to the stimulation of IFN-gamma production by T cells and exerted a protective effect on mice exposed to a lethal dose of influenza virus as has been previously shown *in vivo* study (Utsunomiya et al., 1997).

Identifying host genes that are critical to SARS-CoV-2 infection may identify new therapeutic targets and help us understand the pathogenesis of COVID-19. The superiority of targeting intracellular molecules rather than viral proteins is that mutations in the viral genome are unlikely to counteract their effects. However, the mechanism by which genes other than HMGB1 regulate viral infection remains to be verified. Many effective drugs work by modulating multiple proteins rather than a single target. Network pharmacology integrates a variety of modern publicly available database resources and collects a full range of biomedical information such as drugs, compounds, genes, and disease targets to build a multilevel complex network of drug-target-disease interactions, to reveal the mechanism of natural compounds from Chinese Medicine that have multiple components and multiple targets in the human body. In this way, network pharmacology is becoming the next paradigm for drug discovery, especially in the field of Chinese medicine (Hopkins, 2008). Based on this network pharmacology, we here to explore possible mechanism by which GA affects for COVID-19 by targeting pivotal inflammatory pathways of the host triggered by SARS-CoV-2.

## Materials and Methods

### GA Targets and COVID-19-Related Genes

We collected potential targets of GA in various databases, including SwissTargetPrediction database (STP, version: 2019, http://www.swisstargetprediction.ch) (Daina et al., 2019), STITCH (version: 5.0, http://stitch.embl.de/) (Szklarczyk et al., 2016), PharmMapper (version: 2017, http://www.lilab-ecust.cn/pharmmapper/) (Wang et al., 2017), and GEO Dataset (GSE85871 and GSE67504) (Hsiang et al., 2015; Lv et al., 2017), those differentially expressed genes (DEGs) from gene expression profiles in traditional Chinese medicine components (GA) were regarded as potential effector targets as well. DEGs for GSE85871 and GSE67504 datasets were analyzed by GEO2R (https://www.ncbi.nlm.nih.gov/geo/geo2r/). In GEO2R, the Benjamini and Hochberg false discovery rate (FDR) method is selected by default and adj-*p*-value significance level cut-off is 0.05.

COVID-19-related targets were gathered from DEGs by analyzing available transcriptomic RNA-seq COVID-19 data (GSE147507, https://www.ncbi.nlm.nih.gov/geo) (Blanco-Melo et al., 2020), it contains three infected primary lung epithelial samples and three uninfected samples. Screening criteria for DEGs were FDR < 0.05 and log2FC (absolute)  ≥1, and genes that met the inclusion criteria will be used in further analysis (Islam et al., 2020). The common gene identification between GA targets and COVID-19-related targets were obtained using the online tool (http://bioinformatics.psb.ugent.be/webtools/Venn/) and FunRich (http://www.funrich.org/) (Pathan et al., 2015).

### Protein-Protein Interaction Analysis and Network Construction

Common genes were inserted in String database (version 11.0, https://string-db.org/) (Szklarczyk et al., 2019) for generating PPI network. Minimum required interaction score was medium confidence (0.4). The PPI results were analyzed and visualized through Cytoscape (version: 3.8.1, https://cytoscape.org/) (Shannon et al., 2003). The cytoHubba software (http://apps.cytoscape.org/apps/cytohubba) (Chin et al., 2014) and CentiScaPe software (version 2.2, http://apps.cytoscape.org/apps/centiscape) (Scardoni et al., 2009) plugins in Cytoscape were used to performed network topology analysis.

### Gene Ontology and Pathway Enrichment Analysis

We used the ClueGO (version 2.5.4) (Mlecnik et al., 2019) and CluePedia (version 1.5.4) (Bindea et al., 2013) to perform Wiki and KEGG pathway enrichment analysis of GA targets. We chose two-sided hypergeometric test and bonferroni step down for *p* value correction (*p* < 0.05). GO terms and all the pathways enrichment including COVID-19 related gene sets and BioCarta databases were also obtained through Enrichr (https://maayanlab.cloud/Enrichr/) (Kuleshov et al., 2016) for the common genes between GA and COVID-19. Enrichr performed Fisher exact test for random gene sets and adj.*p*.value < 0.05 were regarded as statistically significant.

### Analysis of TF (Transcription factor)-Gene Interactions and TF-miRNA Co-regulatory Network

NetworkAnalyst tool (version 3.0, https://www.networkanalyst.ca/) (Zhou et al., 2019) was used to evaluate the TF-gene interplay with common genes between GA and COVID-19 related targets. The basic data for the TF-gene interplay network was acquired from the ENCODE database (https://www.encodeproject.org/). Only peak intensity signal <500 and the predicted regulatory potential score <1 were used (by using BETA Minus algorithm) (Wang et al., 2013). Regarding TF-miRNA co-regulation network, the interplays for TF-miRNA co-regulation were collected from the RegNetwork repository database (version 2019, http://www.regnetworkweb.org/) (Liu et al., 2015) and we visualized it by using NetworkAnalyst. The current network will help to disclose miRNAs and TFs that regulated the shared genes at the post-transcriptional and transcriptional levels.

### Inferring Upstream Pathway Activity

Extracting the activity of signal transduction pathways from transcriptome data is important for inferring the mechanism origin of abnormal transcriptome regulation. So we used SPEED2 (https://speed2.sys-bio.net/) (Rydenfelt et al., 2020) to infer upstream pathway activity from common genes between GA and COVID-19. When providing a list of genes, the Web server allows inferences about signaling pathways that may cause these genes to be dysregulated. We tested if average gene rank was shifted away from 0 using the Bates distribution on [−1, 1].

## Results

### Target Identification of GA

**FIGURE 1:**
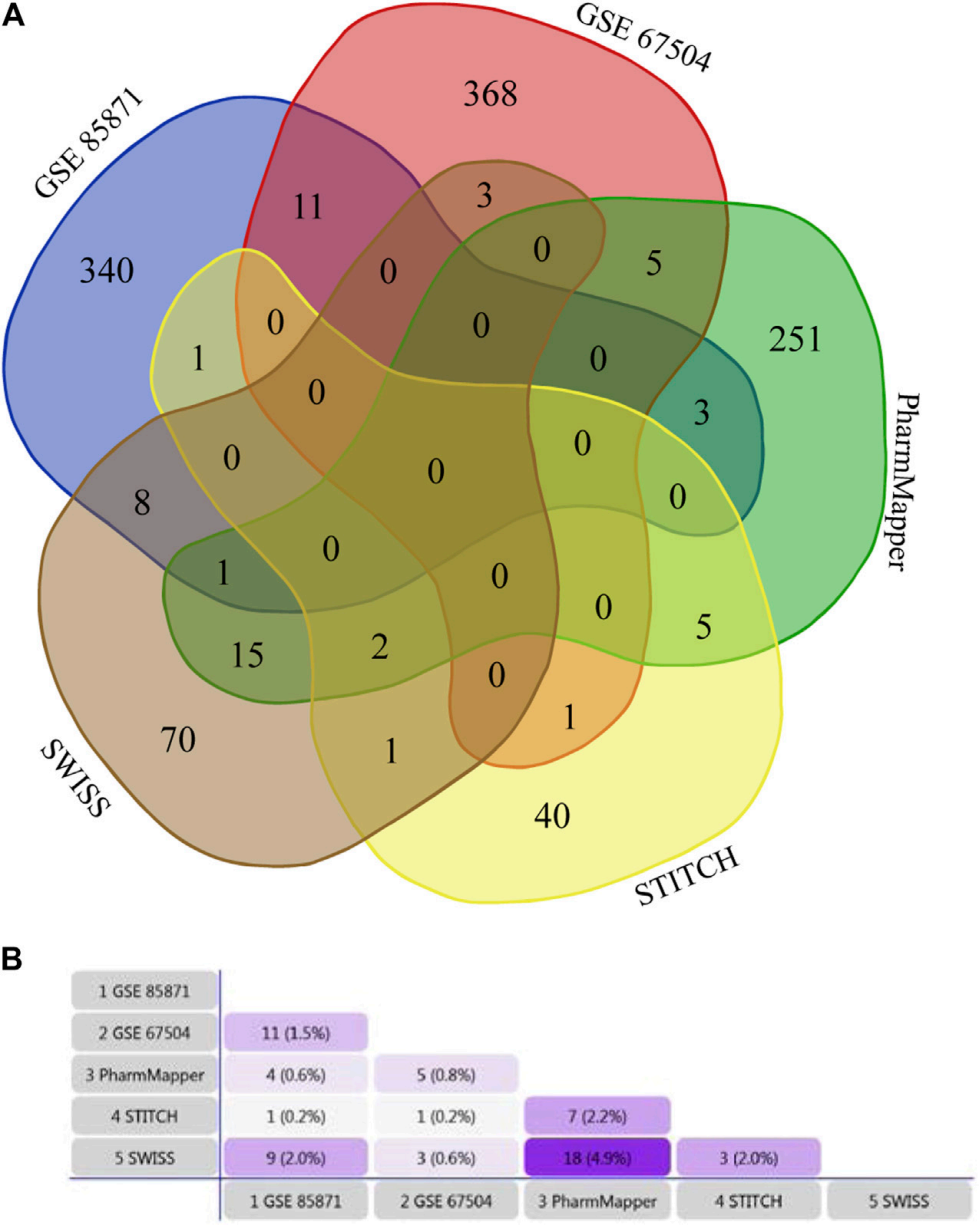
**(A)** Venn diagram based on Draw Venn Diagram Tool, showing the overlapping relationship of GA potential targets from five datasets. **(B)** Venn diagram based on FunRich Tool, showing the constituent ratio of GA potential targets from five datasets.

100 potential targets were predicted from SWISS, 50 from STITCH, and 283 from PharmMapper. 364 DEGs were identified from GSE85871, and 389 DEGs from GSE67504 (Supplementary Table S1). Therefore, 1,125 unique potential targets of GA were scratched together after the removal of duplications. The GA targets of each dataset were shown in Figure 1.

### Pathway Enrichment Analysis of GA Targets

**FIGURE 2:**
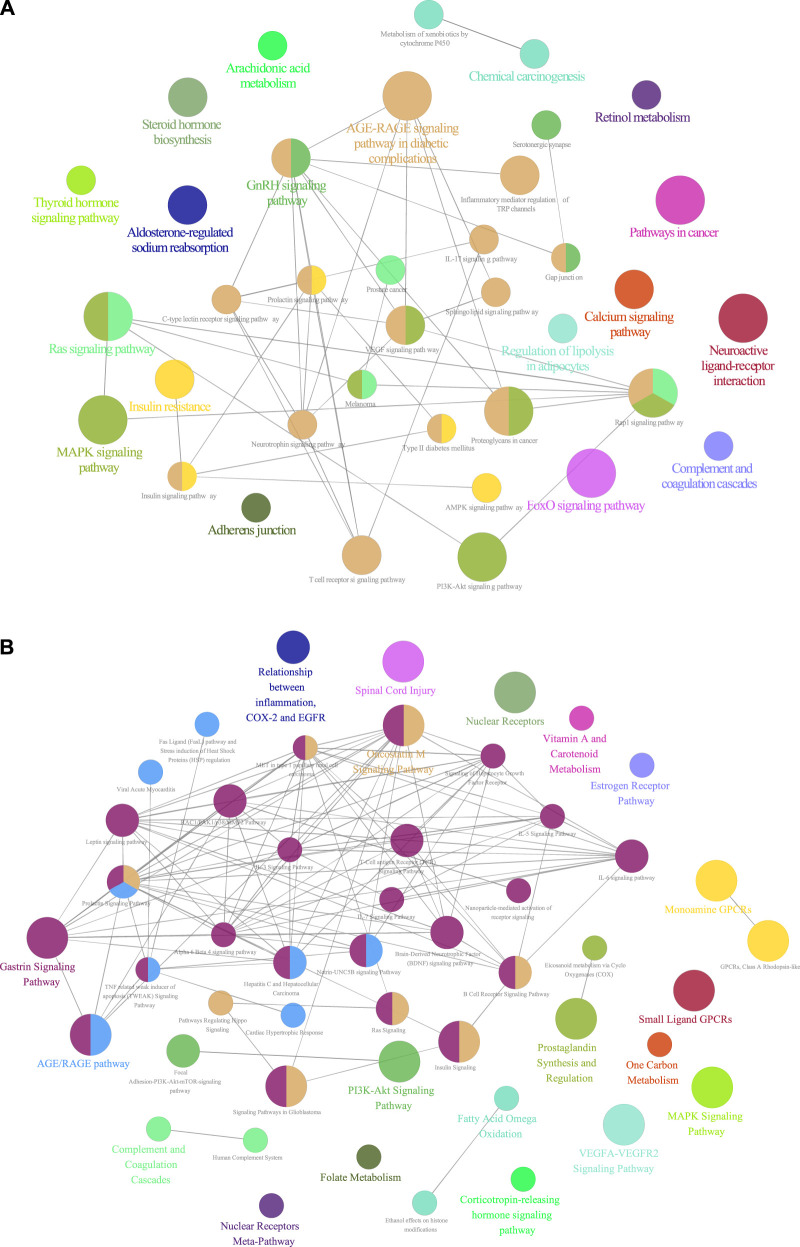
**(A)** KEGG pathways enrichment analysis of GA targets (*p* values < 0.05) **(B)** Wiki pathways enrichment analysis of GA targets (*p* values < 0.05).

KEGG pathway enrichment analysis showed that GA targets involved in 37 pathways. Those targets were meaningfully enriched in multiple pathways such as RAS, MAPK, PI3K-Akt, VEGF, IL-17, and T cell receptor signaling pathways (Figure 2A and Supplementary Table S2). The 49 enriched Wiki pathways were involved in estrogen receptor, MAPK, TNF related weak inducer of apoptosis, IL-7, IL-6, IL-3, B Cell receptor signaling pathway, signaling of hepatocyte growth factor receptor, and PI3K-Akt-mTOR-signaling pathways (Figure 2B, Supplementary Table S3).

### Protein-Protein Interaction and Network Analysis

**FIGURE 3:**
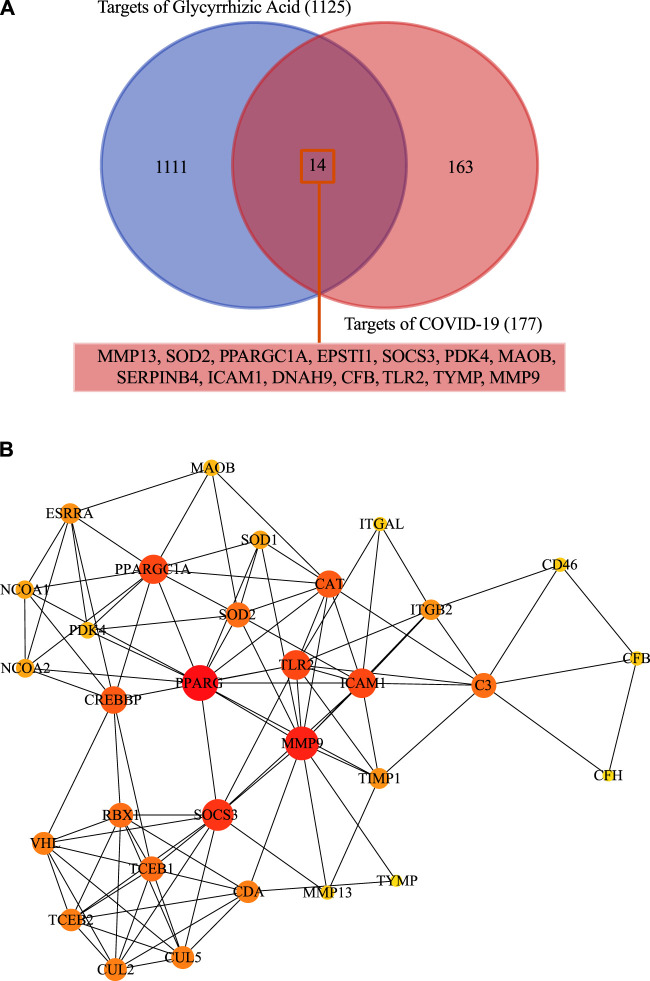
**(A)** Fourteen shared targets were found common from the 117 DEGs of COVID-19 and 1,125 targets of GA. **(B)** Protein-protein interactions network and network topology analysis. Deeper colors represent higher degree values.

**FIGURE 4:**
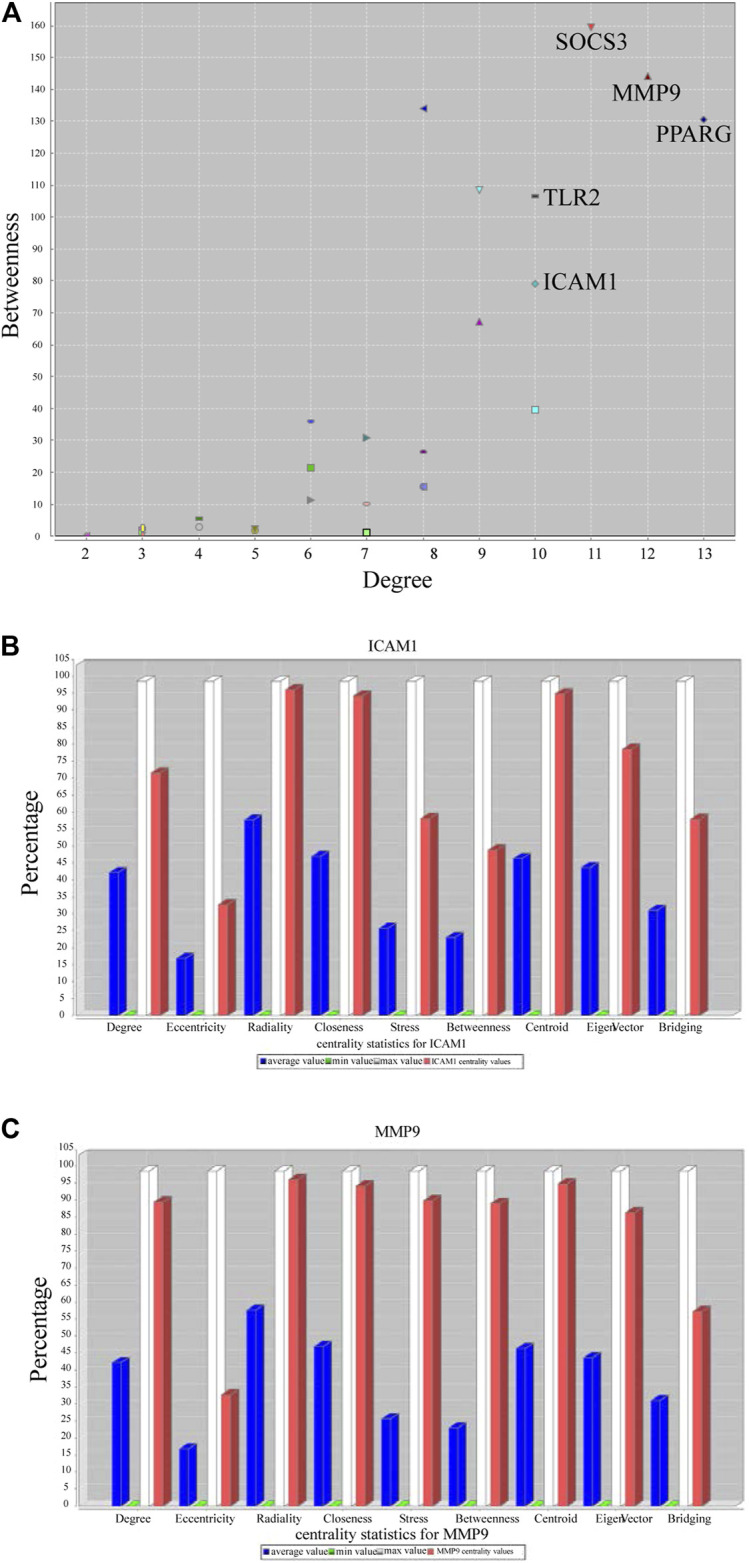
Topological result of GA targets on COVID-19. **(A)** The ordinate indicates the degree, and the abscissa indicates the degree of betweenness **(B)** Network topology analysis parameter of ICAM1. **(C)** Network topology analysis information of MMP9.

We screened the GSE147507 dataset to identify 177 COVID-19-associated genes. Compared with GA target, venn diagrams revealed 14 common targets against COVID-19 (Figure 3A). The shared targets were loaded in String and the documents generated by its analysis were re-introduced into Cytoscape for further network topology analysis and visual representation (Figure 3B, Supplementary Table S4). Among those common targets, ICAM1, MMP9,TLR2, and SOCS3 had higher degree values (Figure 4).

### Gene Annotation of Common Targets

**FIGURE 5:**
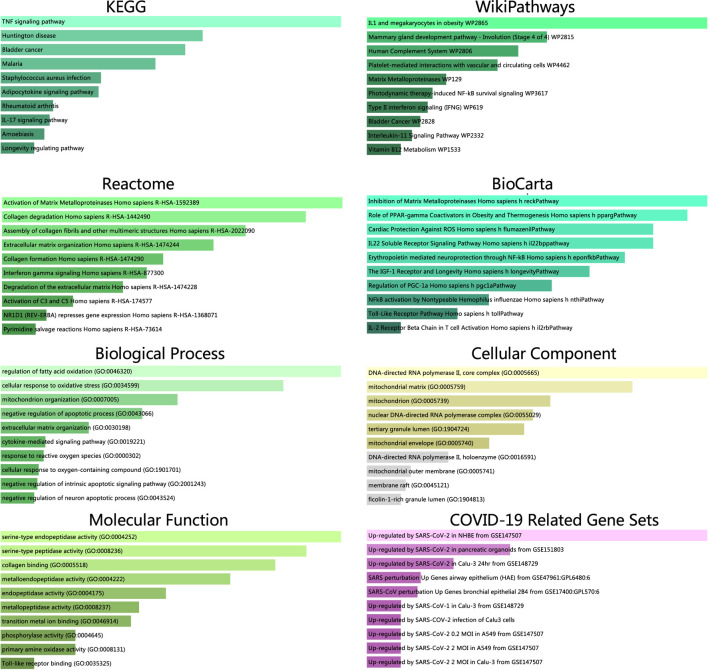
Pathway enrichment analysis from KEGG, WikiPathways, Reactome and BioCarta databases. GO terms enrichment analysis including biological process, molecular function and cellular component aspects. All the results along with COVID-19 related gene sets were identified through the *p* value.

We identified 14 shared targets of GA related to COVID-19 (MMP13, SOD2, PPARGC1A, EPSTI1, SOCS3, PDK4, MAOB, SERPINB4, ICAM1, DNAH9, CFB, TLR2, TYMP, MMP9). Enrichr tool was used to perform the analysis of gene set enrichment including GO terms, pathways and COVID-19 related gene sets. The GO enrichment study illustrated the three parts (biological process, molecular functions, and cellular component) of the top 10 GO terms. The pathway enrichment study illustrated the KEGG, WikiPathways, Reactome, and BioCarta of the top 10 pathway terms (Figure 5 and Supplementary Table S5).

### TF-Gene Interplays

**FIGURE 6:**
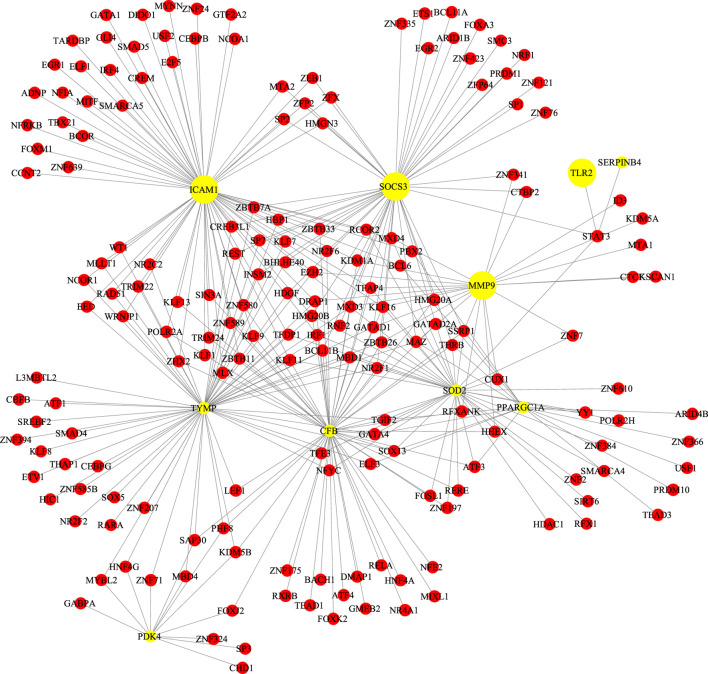
Network for transcription factor gene interplay with shared targets.

TF-gene interplays were constructed by NetworkAnalyst tool. The selected targets of the previous analysis were matched to the corresponding molecular interaction database. Subnetworks with at least three nodes were listed and visualized below (Figure 6). The network consisted of 189 nodes and 325 edges. Targets in the T-F network were associated with regulating immunity and anti-inflammatory effect. ICAM1 was regulated by 69 TF genes and MMP9 was regulated by 22 TF genes.

### TF-miRNA Co-regulation Network

**FIGURE 7:**
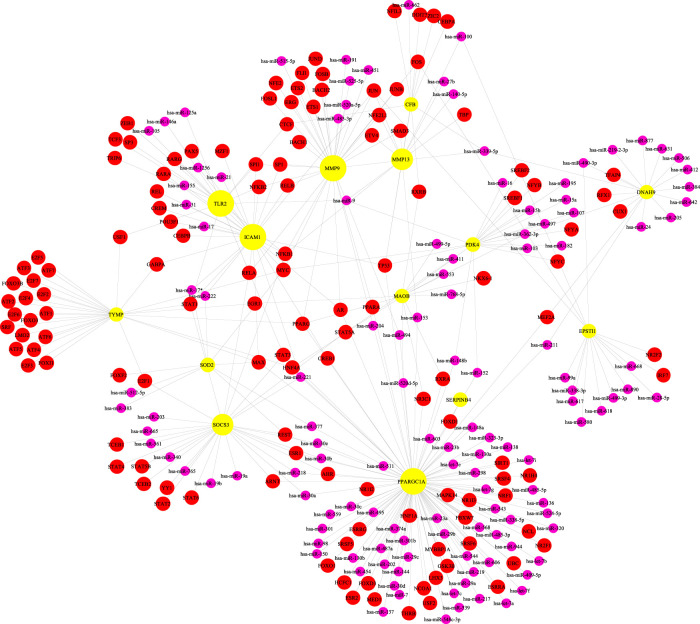
The network presents the transcription factor-miRNA coregulatory network. The nodes in yellow are the shared targets, a purple node displays miRNA and other nodes in red color represent transcription factor -genes.

TF-miRNA co-regulation network was performed by NetworkAnalyst tool. Subnetworks with at least three nodes were shown below (Figure 7). The network consisted of 270 nodes and 343 edges. This interplay may be responsible for regulating the expression of the above shared targets. The degree of MMP9, ICAM1 and SOCS3 were 35, 31 and 29, respectively.

### Upstream Pathway Activity

**FIGURE 8:**
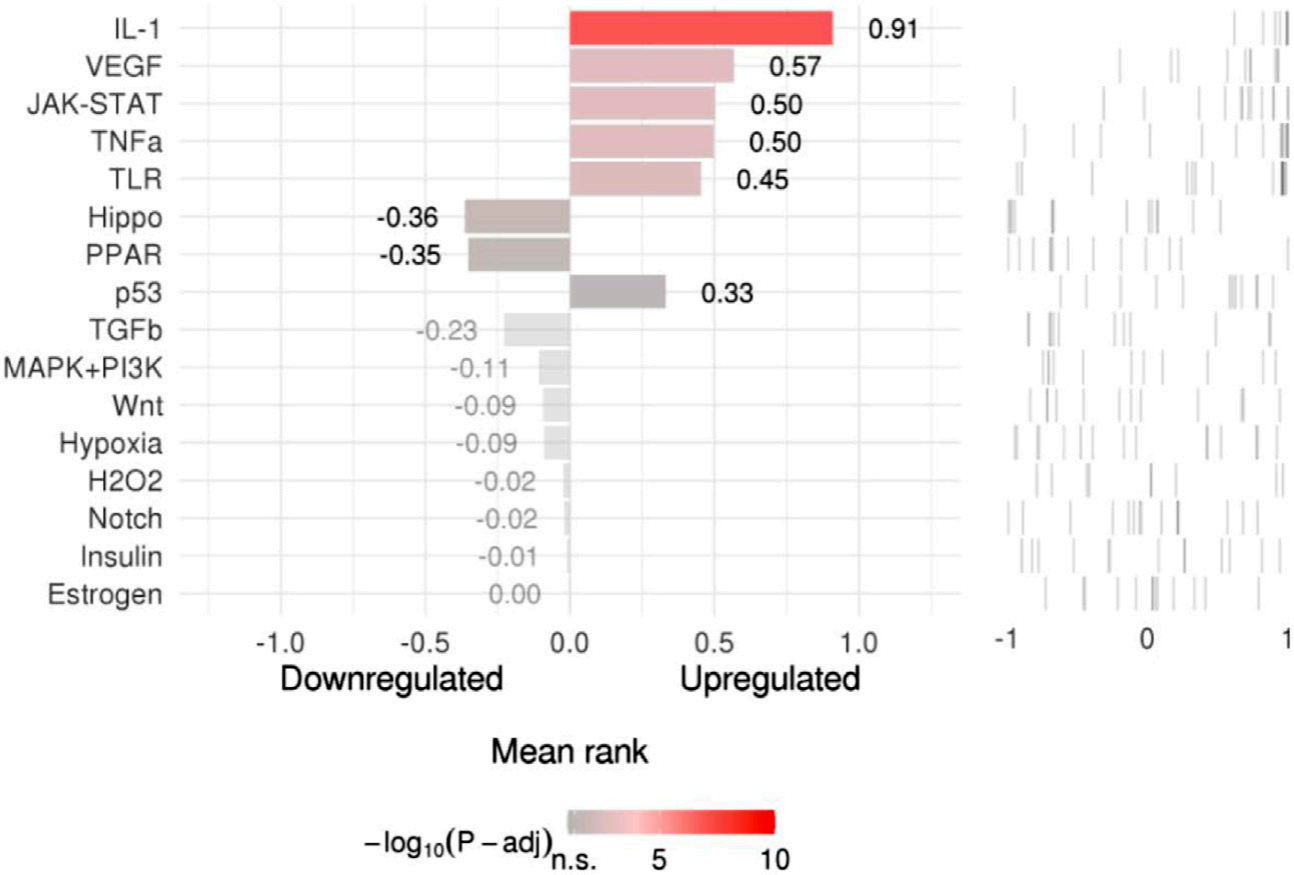
Pathway activity ranking.

SPEED2 offered an easy method to score signaling activity for sets of dysregulated genes gained from transcriptome analysis. To infer the upstream pathway activity from common targets, we evaluated the consistently up and down-regulated genes after pathway perturbation, and then we scored the genes. The ranked lists were determined by *p*-value, the more significant genes are up-regulated, the higher the ranking, the most significantly down-regulated genes matched to the bottom of the list. Colors showed FDR-adjusted *p*-values (Figure 8 and Supplementary Table S6).

## Discussion

The results of pathway enrichment analysis showed that the KEGG and WIKI pathways of GA targets were mainly involved in multiple inflammation- and immune-related signaling pathways, including PI3K- AKT, IL-17, and MAPK signaling pathways, as well as complement and coagulation cascades and inflammatory mediator regulation. Activation of the coagulation pathway during the immune response to viral infection leads to the overproduction of pro-inflammatory cytokines, which leads to multiple organ damage. The interplay between inflammation and coagulation may contribute to COVID-19 cytokine storm (Jose and Manuel, 2020). The sustained inflammatory response caused by SARS-CoV-2 can cause cytokine storms that lead to activation of coagulation and complement cascades and this may trigger an acute respiratory distress syndrome (ARDS) (Wang et al., 2020). Research revealed that GA is a selective inhibitor of thrombin, thus it is possible that changes in this pathway impact SARS-CoV-2 infection process, as has been previously shown for antithrombotic effect (Mendes-Silva et al., 2003).

GA is a triterpene saponin with various pharmacological effects. It has been found to inhibit viral replication of SARS-CoV *in vitro*, GA has been found to inhibit adsorption and penetration of the early stages of the viral replication (Cinatl et al., 2003). Regarding the mechanism underlying the action, nitrous oxide may be responsible for inhibiting viral replication. Other studies have shown that inhibiting the TLR-4/NF-κB signaling pathway may be a mechanism of GA action, in which proinflammatory cytokines are inhibited during the initial phase of the inflammatory response (Lee et al., 2019). GA inhibited TLR2, which inactivated TLR signaling pathway to reduce lipopolysaccharide-induced acute lung injury (Kong et al., 2019). One study indicated that GA inhibits the increase in inflammatory factors in ALI by regulating the PI3K/AKT/mTOR pathway (Qu et al., 2019). GA regulated the expression of inflammatory cytokines and CXCL-2 by blocking the IL-17/STAT3 pathway, thereby inhibiting neutrophil airway inflammation (Kim et al., 2020). GA alleviated the inflammation by reducing neutrophil and macrophage infiltration in lung tissue and the multiple cytokines including HMGB1, TNF-α, IL-1β, IL-6, and IL-8 in serum and lung tissue (Yao and Sun, 2019). Because reactive oxygen species (ROS) play a key role in the inflammatory response, antioxidants may also be useful against cytokine storms caused by infections (Liu et al., 2016), and GA has a pronounced ROS quenching activity, blocking the cascade of incidents including MAPK pathway and cell death (Farrukh et al., 2015). Furthermore, GA itself has the properties of anti-inflammatory via TLR4 antagonism so as to compensate for the reduced protection of ACE2 downregulation (Murck, 2020). These mutually dependent results indicated that GA may have therapeutic benefits for COVID-19 by targeting several core inflammatory pathways of the host triggered by SARS-CoV-2, as has been previously shown for anti-inflammation effect in ARDS and ALI.

Regarding the key targets of GA for COVID-19. Compared with the analysis of SARS-CoV-2 affected lung tissue samples, we obtained 14 common targets of GA against COVID-19 (MMP13, SOD2, PPARGC1A, EPSTI1, SOCS3, PDK4, MAOB, SERPINB4, ICAM1, DNAH9, CFB, TLR2, TYMP, MMP9), those overlapping targets of GA-related targets and SARS-CoV-2 DEGs were considered as potential targets, the results of protein-protein interaction and network analysis showed that ICAM1, MMP9, TLR2, and SOCS3 had higher degree values, which may be core targets of GA for COVID-19 treatment. Then we picked those 14 targets into Enrichr tool to perform gene annotation.

A basic element in our study was harnessing common targets between GA and COVID-19 to develop a more extended therapeutic hypothesis. Our study suggests that ICAM1, MMP9, TLR2, and SOCS3 are a promising intervention target: SARS-CoV-2 infection aggravates the endothelin induction in multiple organs of COVID-19 patients via viral involvement in the host inflammatory and immune response, and the presence of elements within endothelial cells and the gathering of inflammatory cells (Varga et al., 2020). The results of clinical research suggested that in severe COVID-19 patients, intercellular adhesion molecule 1 (ICAM-1), also known as cluster of differentiation 54 (CD54), was significantly elevated, but it was reduced during the recovery period. The increased expression of endothelial cell adhesion molecules like ICAM1 is related to the severity of COVID-19 and may lead to coagulation dysfunction (Tong et al., 2020). Activated endothelial cells contribute to the expression of ICAM-1, which may attract leukocytes in addition to transmitting intracellular signals, resulting in a sustained pro-inflammatory state. The continuing inflammatory signals of these adhesion molecules can also cause thrombotic events (Nagashima et al., 2020).

The hub node MMP9 is an immunogenic protease related to cell-matrix degradation. It was initially thought that the main function of matrix metalloproteinases (MMPs) was to degrade various components of the extracellular matrix (ECM), but recent studies have shown that they were important regulators of signal transduction networks in extracellular tissues (Loffek et al., 2011). Macrophages produce many inflammation-associated molecules, released by MMPs, such as adhesion molecules and cytokines (Li K. et al., 2020). MMP9 producing mononuclear cells enable T cells to invade the vascular wall and trigger vasculitis. The production of proinflammatory cytokines and vascular inflammation was mediated through TLRs, NLRs and other pattern recognition receptors. Particularly, viral particles of SARS-CoV-2 infection may contribute to TLRs activation directly or indirectly (Biswas and Khan, 2020). SARS-CoV-2-mediated TLRs activation was blamed for the later activation of the extrinsic coagulation pathway, which ultimately resulted in thrombosis.

In GO BP analysis, response to reactive oxygen species (ROS) was significantly enriched. Interestingly, it has been demonstrated that GA exerts an anti-inflammatory effect by reducing ROS production in neutrophils (Akamatsu et al., 1991). The current cellular components analysis of GO enrichment displayed the connection of MMP9 with function of lumens releasing tertiary granules. We found that the MMP9 was related to serine protease activities in molecular function aspect. Those results were consistent with previous researches (Cassatella et al., 2019). TLR-2, TLR-3, and TLR-4 activation by SARS-CoV-2 triggered the release procedure of IL-1β. The binding of it to TLRs contributed to the release of pro-IL-1β, the activation of inflammatory, and the production of mature IL-1β. What‘s more, plasmin activated MMP9 in monocytes and macrophages level, all of these further responds with TLR9 signaling to induce the generation of TNF that caused the expansion of cytokine storm (Gomez-Salinero and Rafii, 2017).

Regarding the pathway enrichment analysis, the present study shows that the IL-17, IL-6, TNF-α, and IFN signals, complement system, and growth factor receptor signaling were major pathways by which GA targets COVID-19. In ARDS, by stimulating the production of pro-inflammatory mediators, IL-17 increased the damage to lung parenchyma through the recruitment of maladaptive neutrophils (Muir et al., 2016). What’s more, the addition of exogenous IL-17 further aggravated the production of TNF, IL-1β, IL-6, and CXCL2 induced by LPS. These analyses showed that IL-17 can be used not only as a biomarker of disease severity but also as a potential therapeutic target to reduce SARS-CoV-2, particularly the damage to the lungs (Pacha et al., 2020). IL-6 levels are associated with respiratory failure and poor prognosis. Most studies hold the view of blocking IL-6 and other signals and cytokines in the early period was beneficial for specific and adverse immune responses in COVID-19 patients (Copaescu et al., 2020). The complement system plays an important role in the innate immune response to all viruses including SARS-CoV-2, which is also an attractive therapeutic target in COVID-19 by decreasing the severity and end-organ outcomes of this kind of response (Risitano et al., 2020). The formation of IFNs is a universal host response after viral infection. IFN signals are the primary and front line signals, which launch a fast antiviral response in host cells (Catanzaro et al., 2020). Well known that TNF-α, performing as a megaphone of inflammation, is crucial in nearly all kinds of acute inflammatory responses, including COVID-19. TNF-α serum levels in SARS-CoV-2 infection served as independent and sensitive predictors of disorders severity and mortality (Del Valle et al., 2020). Inhibiting the growth factor receptor signaling pathway may prevent SARS-CoV-2 replication, which suggested practical strategies for COVID-19 treatment (Klann et al., 2020). From those KEGG and WIKI pathway terms, results showed that targets of GA were significantly enriched in these pathways. Therefore, in the future clinical management of severe COVID-19 cases, the regulations of the mechanism behind the inflammation immune dysregulation of GA against SARS-CoV-2 might give us therapy clues for preventing the transition from mild to severe stages (Li H. et al., 2020).

TF gene interplay was gained with the 14 shared targets. The TF genes served as regulators in accordance with genetic expressions. In this study, we found that ICAM1, MMP9, and TLR2 showed a high interplay degree with other TF genes. The degree of ICAM1 in this network is 69. Among the regulators in the TF gene interaction network, STAT3 and MMP9 together with TLR2 have significant interaction. STAT3 transcriptionally induces suppressor both SOCS1 and SOCS3, which later inhibit JAKs activity, and the viral components of SARS-CoV-2 induce compensatory hyperactivation of STAT3, which may lead to thrombosis, so an abnormal STAT signaling pathway is considerable in SARS-CoV-2 infection (Matsuyama et al., 2020). This pathological phenomenon toward STAT3 may contribute to the clinical manifestations most common with COVID-19 patients in the hospital, such as coagulopathy, proinflammatory status, profibrotic condition, and T cell lymphopenia. Based on the shared targets, we generated the TF-miRNA co-regulatory network that involved the interaction of miRNAs and TF genes. We found that PPARGC1A had the highest degree. The protein of this gene is a transcriptional co-activator that can regulate genes related to energy metabolism, so that the protein interacts with a variety of transcription factors, and may also participate in the control of blood pressure and the homeostasis of cellular cholesterol. The regulation of TF genes is finished by binding with targeted genes and miRNAs, they are able to regulate gene expression through mRNA degradation (Zhang et al., 2015). At last, we scored signaling activity for dysregulated genes obtained from the above network analysis. We found the majority of common targets between GA and COVID-19 to be upregulated in the IL-1, VEGF, JAK-STAT, TNFα, and TLR pathway families. This was consistent with our other research conclusions.

Taken together, our integrative study identified essential targets of GA in COVID-19. The discovery of vital signal pathways that reformed during SARS-CoV-2 infection may help reveal the most meaningful molecular protein interaction that mediates viral infections (Catanzaro et al., 2020). Currently, a limited number of vaccines have been approved and the process of vaccination is going or being planned in few countries (Rabaan et al., 2020). Potential effective therapeutic agents are desperately needed in future clinical practice. Targeting pathways triggered by SARS-CoV-2 infection is an appropriate strategy for the treatment of COVID-19. It also inhibits cytopathic effects. Fortunately, GA may exert a therapeutic function in COVID-19 by suppressing SARS-CoV-2 infection through its combination of antioxidative, antiviral, and anti-inflammatory effects, especially activation of the immune system.

## Conclusion

We used network pharmacology and bioinformatic analysis to determine the pharmacological mechanism by which GA affects COVID-19. GA may be a suitable molecular drug for ameliorating excessive inflammation triggered by SARS-CoV-2 through inhibition of the IL-17, IL-6, and TNF-α signaling pathways. Although solid proof of GA application on COVID-19 remains unclear, the anti-inflammatory and antiviral effects of GA have been proven in SARS-CoV related and other numerous studies. We found that targeting critical inflammatory pathways of SARS-CoV-2 infection may provide a suitable strategy to tackle the cytokine storms produced by COVID-19. The natural product GA is likely to be beneficial for the patients. However, the study of mechanisms should be based on the confirmed effect of GA on COVID-19 and SARS-CoV-2. These results need immediate further strategies like further experimental validation. This will contribute a lot for GA to be a successful molecular drug against the COVID-19 pandemic.

## Data Availability

The original contributions presented in the study are included in the article/Supplementary Material, further inquiries can be directed to the corresponding authors.

## Supplementary Material

SUPPLEMENTARY TABLE 1 GA targets from five databases.

SUPPLEMENTARY TABLE 2 KEGG pathways enrichment result of GA targets.

SUPPLEMENTARY TABLE 3 WikiPathways enrichment result of GA targets.

SUPPLEMENTARY TABLE 4 Protein-protein interaction analysis network of common targets.

SUPPLEMENTARY TABLE 5 Gene annotation of common targets.

SUPPLEMENTARY TABLE 6 Pathway activity ranking.
